# Supplementary material for: MAGORINO: Magnitude‐only fat fraction and R* 2 estimation with Rician noise modeling
Source: Magn Reson Med. 2022 Nov 2;89(3):1173–92. doi: 10.1002/mrm.29493 (PMC10092287; doi:10.1002/mrm.29493)

Supplementary information

Supplementary Information Table 1 - Summary of models and fitted parameters. All models were initialized using both fat- and water-dominant initializations, as specified in the second column from the right. The objective functions and estimation of $\boldsymbol{f_B}$ varied between methods (i)-(iii). The constant $\boldsymbol{C}$ effectively compensates for reduction in the signal magnitude due to $\boldsymbol{R_2^*}$ decay and chemical shift and avoids the need for empirical manual adjustment of initial values depending on scanner gain, as performed in [30]. *For complex fitting, the Gaussian log likelihood is computed separately for real and imaginary channels before summation.

<table>
<tr><th rowspan="2">Fitting method</th><th rowspan="2">Objective function</th><th rowspan="2">Fitted Parameters</th><th colspan="2">Initialization</th></tr>
<tr><th>$\boldsymbol{\rho_w, \rho_f, R_2^*}$</th><th>$\boldsymbol{f_B}$</th></tr>
<tr><td>i. Gaussian magnitude</td><td>Gaussian log likelihood (Eq 4)</td><td>ρ_w, ρ_f, R_2^*</td><td rowspan="3">Water dominant: $\{\rho_w, \rho_f, R_2^*\} = \{C * \max_t |S_t|, 0.001, 0.1\text{ms}^{-1}\}$
Fat dominant: $\{\rho_w, \rho_f, R_2^*\} = \{0.001, C * \max_t |S_t|, 0.1\text{ms}^{-1}\}$</td><td>-</td></tr>
<tr><td>ii. Rician magnitude</td><td>Rician log likelihood (Eq 4)</td><td>ρ_w, ρ_f, R_2^*</td><td>-</td></tr>
<tr><td>iii. Complex
(f_B estimated)</td><td>Complex Gaussian log likelihood (Eq 8)*</td><td>$\rho_w, \rho_f, R_2^*, f_B$</td><td>$f_B$ = 0</td></tr>
</table>

Supplementary Information Table 2 - Summary of linear regression parameters for multisite phantom dataset (agreement between measured PDFF values and reference values).

	MAGORINO PDFF 1.5T Protocol 1			MAGO PDFF 1.5T Protocol 1		
**Site**	**R^2**	**Slope [95% CI]**	**Intercept [95% CI]**	**R^2**	**Slope [95% CI]**	**Intercept [95% CI]**
**1**	0.999	0.986 [0.964 – 1.010]	0.012 [0.004 – 0.021]	0.999	0.986 [0.964 – 1.007]	0.012 [0.004 – 0.020]
**2**	0.997	1.006 [0.965 – 0.1047]	0.011 [-0.005 – 0.027]	0.997	1.007 [0.964 – 1.050]	0.010 [-0.006 – 0.027]
**3**	1.000	0.984 [0.969 – 1.000]	0.009 [0.003 – 0.015]	1.000	0.983 [0.968 – 0.998]	0.009 [0.003 – 0.015]
**4**	0.990	0.907 [0.836 – 0.977]	0.006 [-0.020 – 0.033]	0.988	0.908 [0.834 – 0.983]	0.006 [-0.022 – 0.035]
**5**	0.999	0.989 [0.973 – 1.006]	0.004 [-0.002 – 0.011]	0.999	0.989 [0.972 – 1.007]	0.004 [-0.003 – 0.011]
**6**	0.998	0.980 [0.946 – 1.014]	0.002 [-0.011 – 0.015]	0.998	0.979 [0.944 – 1.014]	0.002 [-0.011 – 0.015]
	**MAGORINO PDFF 1.5T Protocol 2**			**MAGO PDFF 1.5T Protocol 2**		
**Site**	**R^2**	**Slope [95% CI]**	**Intercept [95% CI]**	**R^2**	**Slope [95% CI]**	**Intercept [95% CI]**
**1**	0.996	0.985 [0.938 – 1.031]	0.020 [0.002 – 0.038]	0.996	0.984 [0.939 – 1.029]	0.019 [0.001 – 0.036]
**2**	0.998	1.003 [0.972 – 1.034]	0.013 [0.001 – 0.025]	0.998	1.002 [0.972 – 1.032]	0.013 [0.002 – 0.025]
**3**	0.938	0.735 [0.592 - 0.878]	0.063 [0.008 – 0.118]	0.938	0.734 [0.593 – 0.875]	0.062 [0.008 – 0.117]
**4**	0.985	0.813 [0.732 - 0.891]	0.031 [-0.001 – 0.062]	0.983	0.810 [0.740 – 0.880]	0.028 [0.001 – 0.055]
**5**	0.887	0.641 [0.468 - 0.813]	0.060 [-0.007 – 0.126]	0.887	0.640 [0.469 – 0.812]	0.060 [-0.006 – 0.125]
**6**	0.997	0.984 [0.945 – 1.023]	0.003 [-0.013 - 0.018]	0.997	0.984 [0.944 – 1.023]	0.002 [-0.013 – 0.018]
	**MAGORINO PDFF 3T Protocol 1**			**MAGO PDFF 3T Protocol 1**		
**Site**	**R^2**	**Slope [95% CI]**	**Intercept [95% CI]**	**R^2**	**Slope [95% CI]**	**Intercept [95% CI]**
**1**	0.998	0.990 [0.960 – 1.020]	0.008 [-0.004 – 0.019]	0.998	0.990 [0.960 – 1.020]	0.008 [-0.004 – 0.019]
**2**	0.999	0.999 [0.976 – 1.022]	0.009 [-0.001 – 0.017]	0.999	0.999 [0.976 – 1.021]	0.009 [0.000 – 0.017]
**3**	0.999	0.993 [0.976 – 1.011]	0.007 [0.000 – 0.013]	0.999	0.993 [0.976 – 1.010]	0.006 [0.000 – 0.013]
**4**	0.997	0.983 [0.946 – 1.020]	0.003 [-0.012 – 0.017]	0.998	0.981 [0.943 – 1.019]	0.002 [-0.012 – 0.017]
**5**	0.998	0.945 [0.911 – 0.979]	0.017 [0.004 – 0.030]	0.998	0.944 [0.911 – 0.977]	0.017 [0.004 – 0.030]
**6**	0.998	0.963 [0.932 – 0.995]	0.001 [-0.012 – 0.013]	0.998	0.963 [0.931 – 0.994]	0.000 [-0.012 – 0.012]
	**MAGORINO PDFF 3T Protocol 2**			**MAGO PDFF 3T Protocol 2**		
**Site**	**R^2**	**Slope [95% CI]**	**Intercept [95% CI]**	**R^2**	**Slope [95% CI]**	**Intercept [95% CI]**
**1**	1.000	0.972 [0.959 – 0.984]	0.010 [0.005 – 0.014]	1.000	0.971 [0.959 – 0.984]	0.009 [0.005 – 0.014]
**2**	1.000	0.976 [0.960 – 0.991]	0.005 [-0.001 – 0.011]	1.000	0.975 [0.960 – 0.991]	0.005 [-0.001 – 0.011]
**3**	0.999	0.975 [0.947 – 1.003]	0.014 [0.003 – 0.025]	0.999	0.975 [0.947 – 1.002]	0.014 [0.004 – 0.025]
**4**	0.999	0.958 [0.934 – 0.981]	0.009 [-0.001 – 0.018]	0.999	0.957 [0.932 – 0.982]	0.008 [-0.001 – 0.018]
**5**	0.996	0.949 [0.905 – 0.993]	0.015 [-0.003 – 0.032]	0.996	0.949 [0.906-0.992]	0.014 [-0.002 – 0.031]
**6**	0.998	0.971 [0.939 – 1.003]	0.004 [-0.008 – 0.017]	0.998	0.969 [0.939-0.999]	0.004 [-0.008 – 0.015]

Supplementary Information Figure S1 – Accuracy of sigma estimation methods over the range of plausible SNR values. The figures show the estimated sigma on the y-axis against the true sigma on the x-axis over a range of values corresponding to the expected SNR at typical clinical field strengths *in vivo* (the SNR ranged from 20 to 70). The plots were generated by simulating and fitting 100 pure water, low R_2* voxels with three different degrees of inhomogeneity in the signal intensities within the ROI (a,b,c).

In (a), note that the fitted sigma estimates (red line) consistently slightly underestimate sigma due to a degree of overfitting. Note also that the degree of overfitting is very consistent, even in inhomogenous voxels. To correct for this overfitting, we used linear regression to calculate and correct the slope of the red line. Having derived this slope, we calculated a correction factor *k*, which could be applied to sigma estimates to produce the ‘corrected fitted sigma’ estimates. To allow a fair estimate of the performance of this correction, the slope was derived on a different set of noise instantiations to those used for testing (i.e. different ‘training’ and ‘test’ simulation data were used). The ‘corrected fitted sigma’ method provides a very accurate estimation on the test dataset.

In (b) and (c), the effect of inhomogeneity on sigma estimates is assessed. The inhomogeneity factor species the proportional difference between the largest and smallest $\boldsymbol{S_0}$ (where $\boldsymbol{S_0} = \boldsymbol{\rho_W} + \boldsymbol{\rho_f}$) in the voxel, with all other values evenly spaces between the largest and smallest values. In a homogenous voxel (a), all methods produce accurate sigma estimates. As the voxel becomes more inhomogenous (b, c) the fitting sigma estimates remain accurate but the ROI-based sigma estimated becomes increasingly biased. The most accurate method is the fitting-based sigma estimate with correction, referred to in the legend as ‘corrected fitted sigma’.

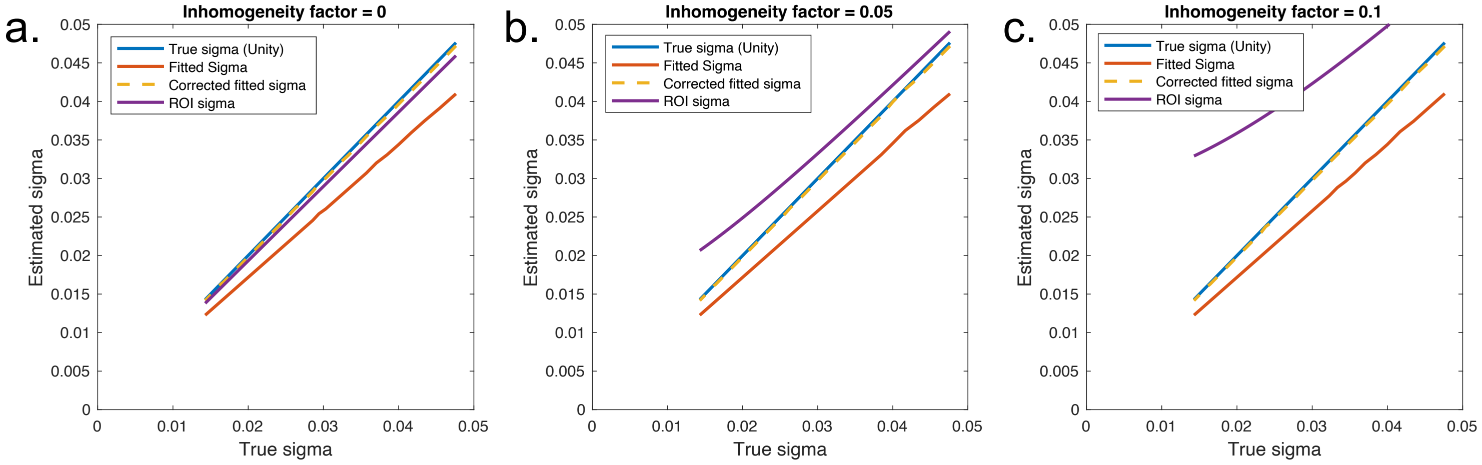

Supplementary Information Figure S2 - Parameter SD for SNR = 60. The plots show the colour-coded standard deviation in PDFF (a-d), R_2* (e-h) and S_0 (i-l) estimates for each combination of PDFF and R_2* values over all simulations, with SNR=60. Note that parameter SD generally increased with increasing R_2* because fat-water swaps become more frequent (as shown in the Figure above). At low R_2*, both Gaussian and Rician magnitude fitting (a,b) show lower PDFF SD than complex fitting (c); the Figures below show that this is because complex fitting does not reach the true (non-swapped) likelihood maximum in every case, resulting in a small positive bias and increase parameter SD. Note that this behaviour is eliminated by fixing $\boldsymbol{f_B}$ (right hand column), although this step is likely to be unrealistic in practice.

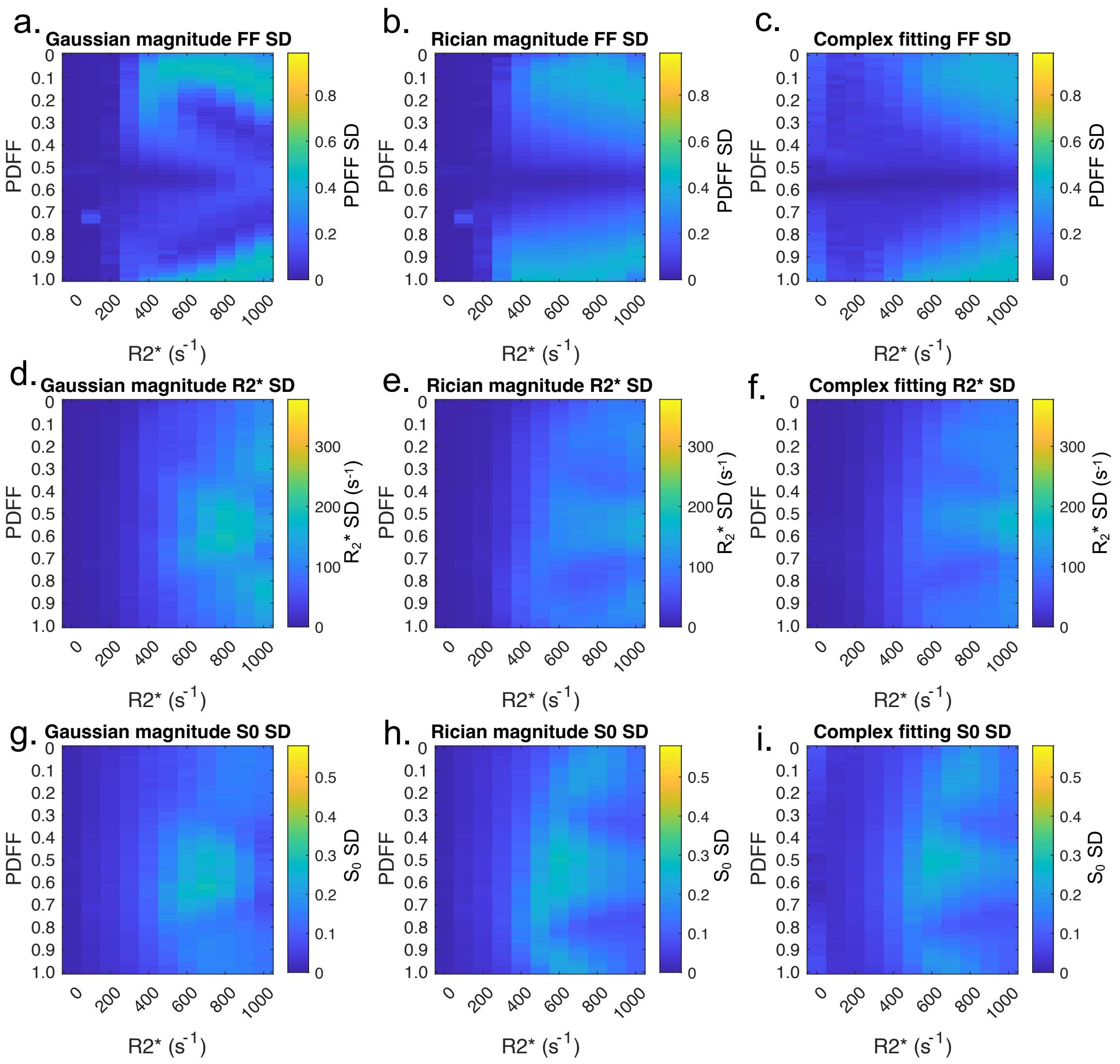

Supplementary Information Figure S3 - Fitting error for SNR = 60. The plots show the grayscale-coded sum of SSE (a-d), 'true SSE' (i.e. SSE calculated relative to the ground truth) (e-h) and estimated noise (SSE / simulated noise SSE) (i-l) for each combination of PDFF and R_2* values over all simulations, with SNR=60. For Gaussian fitting, the 'true SSE' (e) increases substantially at higher R_2* values, indicating overfitting to the noise. This problem is substantially reduced by Rician magnitude fitting and complex fitting. For complex fitting (third column), SSE and noise estimates are highest at low R_2* values because the two-point initialization does not reach the true (non-swapped) likelihood maximum in every case. Note that this behaviour is eliminated by fixing f_B (right hand column), although this step is likely to be unrealistic in practice.

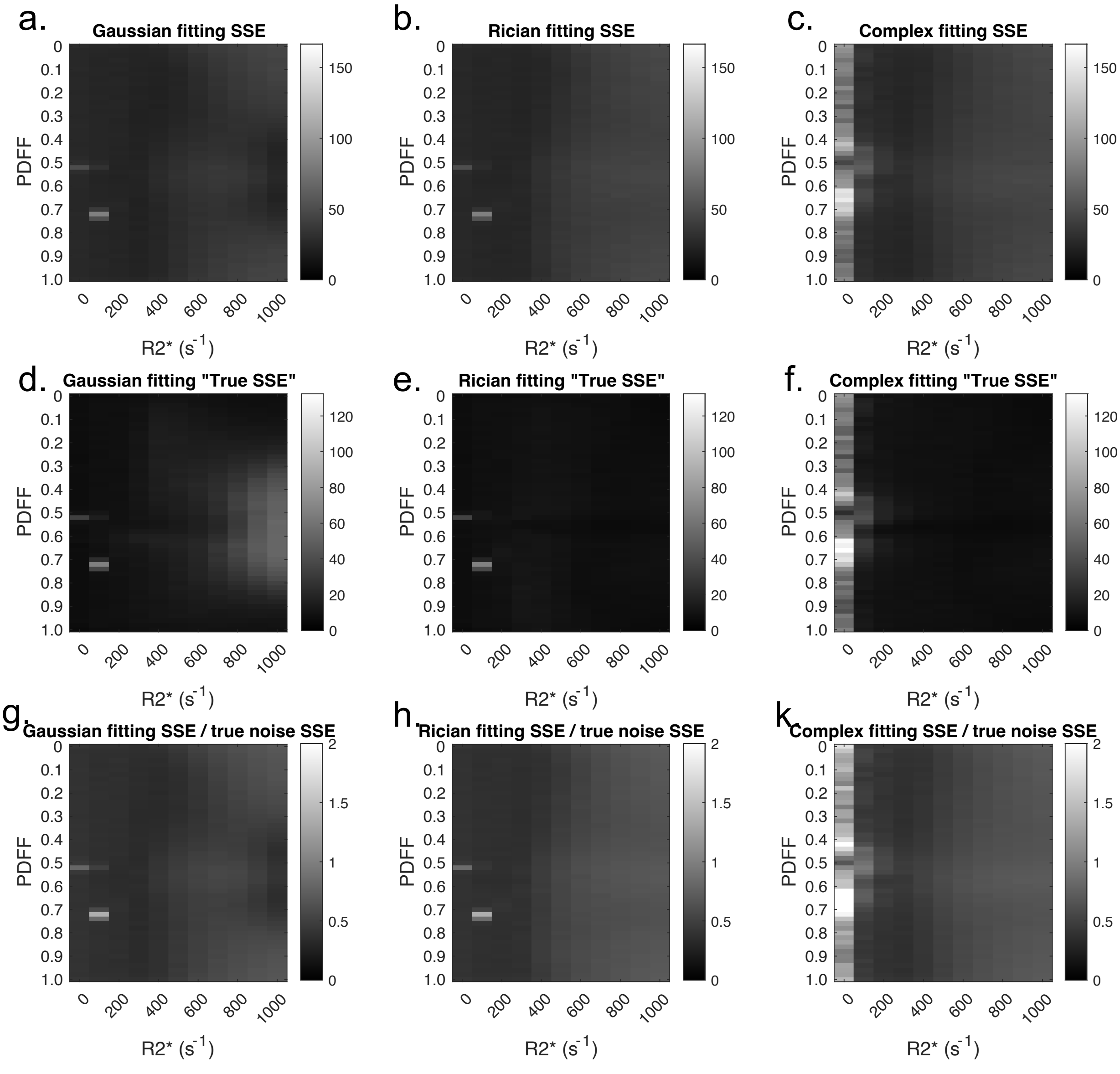

Supplementary Information Figure S4 – Parameter error for SNR = 20. The plots show the colour-coded error in PDFF (a-d), R_2* (e-h) and S_0 (i-l) estimates for each combination of PDFF and R_2* values over all simulations. Note that the benefit of Rician fitting is more pronounced than for SNR = 60.

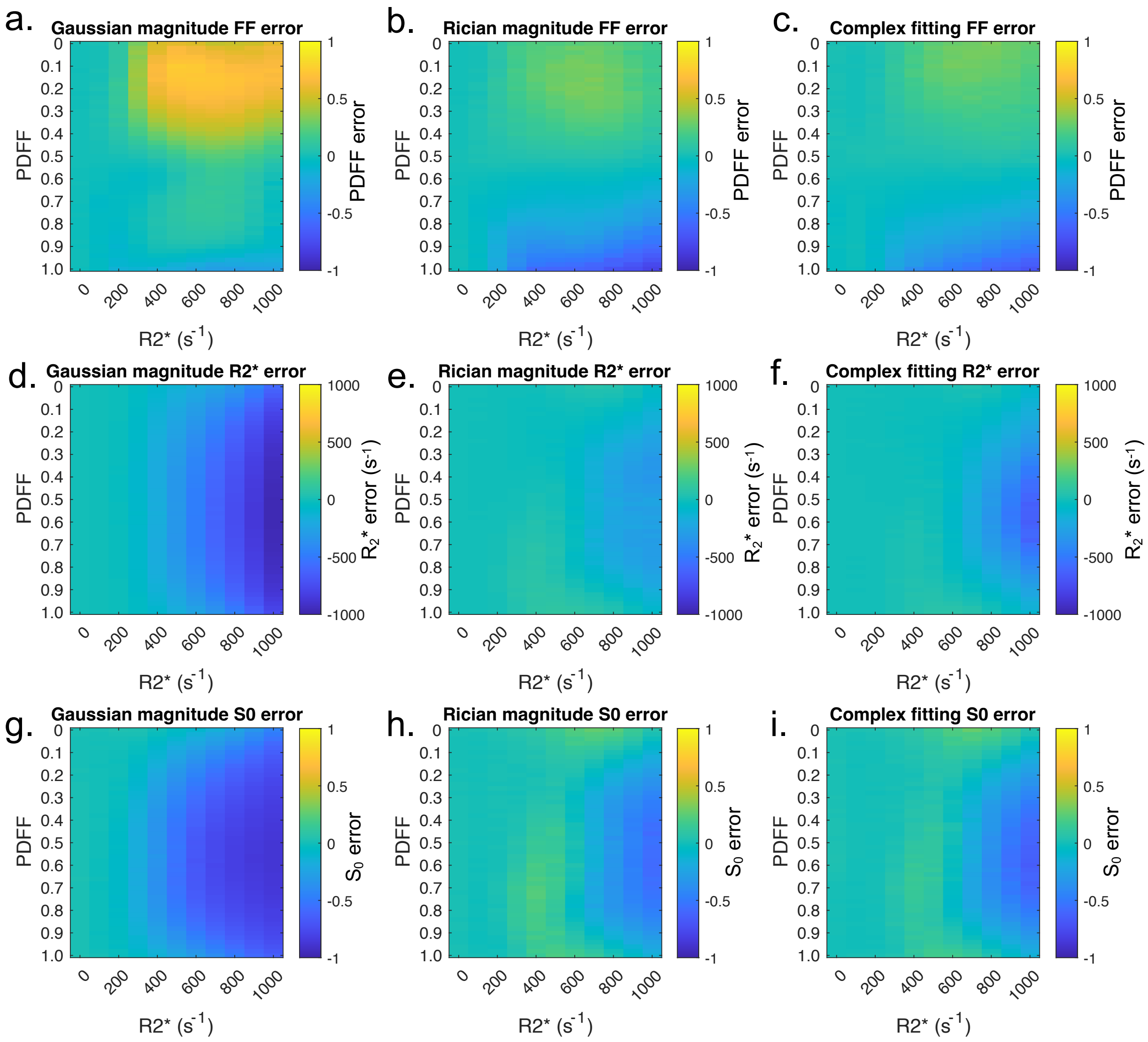

Supplementary Information Figure S5 – MAGORINO is robust to inaccurate sigma estimation. The plots show parameter error maps for PDFF (top row) and for R2* (bottom row) for Gaussian fitting (left column) and for Rician fitting with different sigma accuracies: the second column shows Rician fitting with sigma underestimated, the third column shows Rician fitting with correct sigma, and the right-hand column shows Rician fitting with sigma overestimated. Observe that the effect of the Rician noise model becomes more pronounced from left to right, as the estimated sigma increases (note that Gaussian fitting is equivalent to a sigma assumption of 0). If sigma is underestimated by 30% (second column), the performance becomes closer to Gaussian fitting (MAGO) (left column) than for the correct sigma estimate (third column). If sigma is overestimated by 30% (right-hand column), the differences between MAGO and MAGORINO are exaggerated. Importantly, there is very little deterioration in performance for either under- or overestimated sigma, even with the substantial sigma errors assumed here.

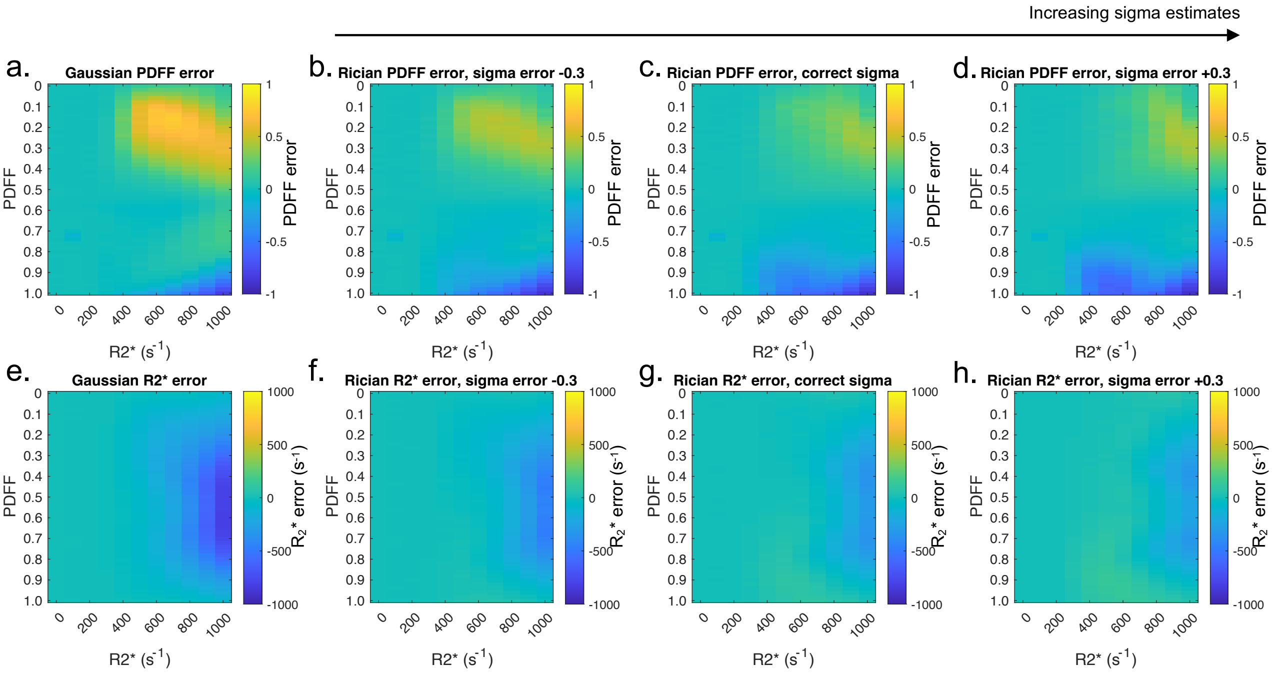

Supplementary Information Figure S6 – Example images of the lower legs (subject 2) for Gaussian fitting (left column) and Rician fitting (middle column). Note that this is a challenging case where complex fitting produced a complete fat-water swap in one leg. Both Gaussian (MAGO) and Rician (MAGORINO) fitting produce satisfactory fat-water separation across the image and good quality R_2* maps, albeit with some non-structured swapping in the subcutaneous fat. As with subject 1, the methods diverge in regions of high R2* / low SNR, which is particularly pronounced in the bone marrow and cortex (red arrows) as well as thie skin. The difference maps (right column) show systematically higher R_2* values in these regions, whilst differences in PDFF can be both positive or negative.

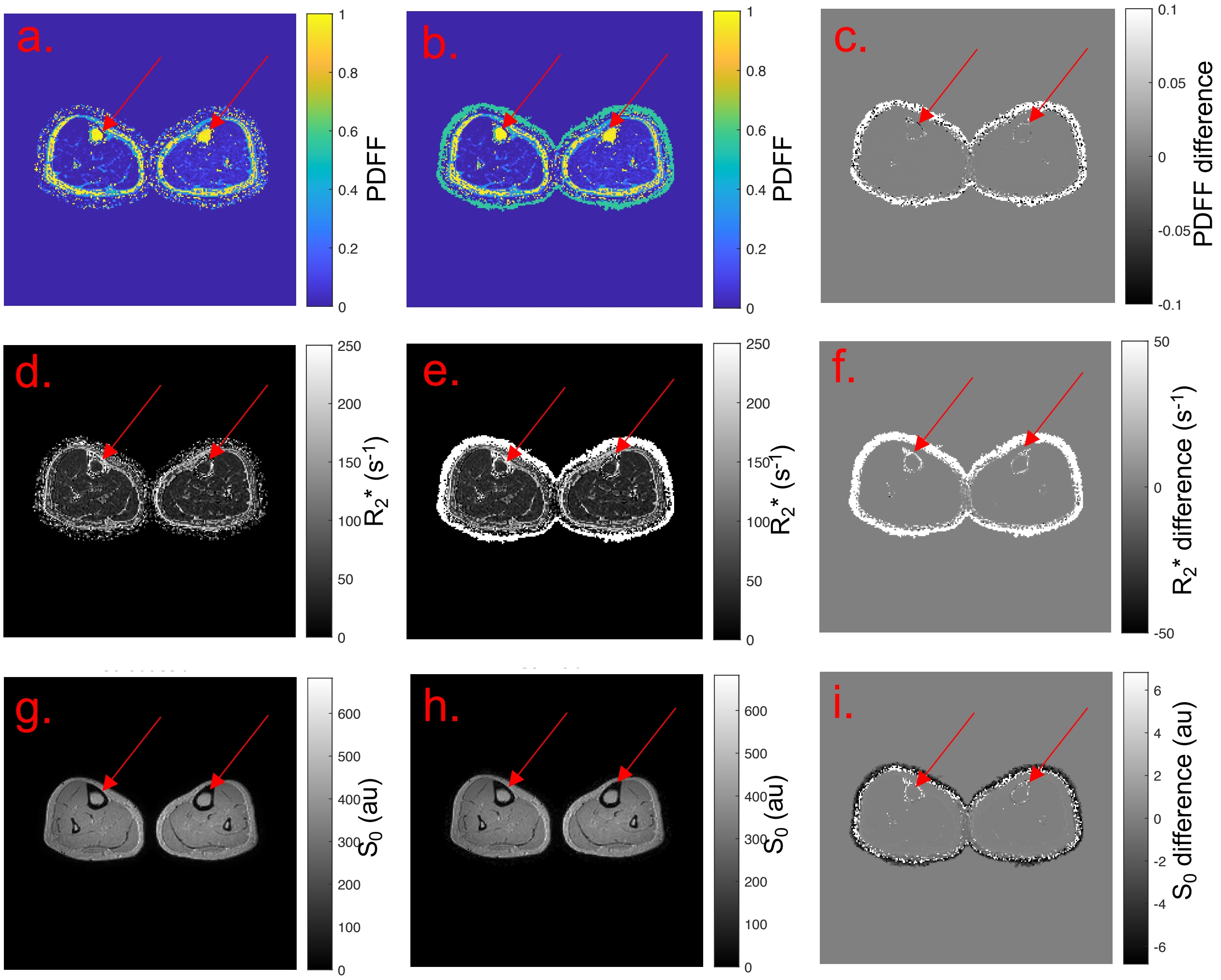

Supplement: Supplementary file 1 — Figure S1. Accuracy of sigma‐estimation methods over the range of plausible SNR values. A–C, Estimated sigma on the y‐axis against the true sigma on the x‐axis over a range of values corresponding to the expected SNR at typical clinical field strengths in vivo (the SNR ranged from 20 to 70). The plots were generated by simulating and fitting 100 pure‐water, low R2* voxels with three different degrees of inhomogeneity in the signal intensities within the region of interest (ROI). In (A), note that the fitted sigma estimates (red line) consistently slightly underestimate sigma due to a degree of overfitting. Note also that the degree of overfitting is very consistent, even in inhomogeneous voxels. To correct for this overfitting, we used linear regression to calculate and correct the slope of the red line. Having derived this slope, we calculated a correction factor k, which could be applied to sigma estimates to produce the “corrected fitted sigma” estimates. To allow a fair estimate of the performance of this correction, the slope was derived on a different set of noise instantiations to those used for testing (ie, different “training” and “test” simulation data were used). The “corrected fitted sigma” method provides a very accurate estimation on the test data set. In (B) and (C), the effect of inhomogeneity on sigma estimates is assessed. The inhomogeneity factor specifies the proportional difference between the largest and smallest S0 (where S0=ρW+ρf) in the voxel, with all other values evenly spaced between the largest and smallest values. In a homogenous voxel (A), all methods produce accurate sigma estimates. As the voxel becomes more inhomogeneous (B, C), the fitting sigma estimates remain accurate but the ROI‐based sigma estimated becomes increasingly biased. The most accurate method is the fitting‐based sigma estimate with correction, referred to in the legend as “corrected fitted sigma” Figure S2. Parameter SD for SNR = 60. The plots show the color‐coded S [file MRM-89-1173-s001.pdf]
